# Supplementary material for: Associations between NK Cells in Different Immune Organs and Cellular SIV DNA and RNA in Regional HLADR− CD4+ T Cells in Chronically SIVmac239-Infected, Treatment-Naïve Rhesus Macaques
Source: Viruses. 2022 Nov 13;14(11):2513. doi: 10.3390/v14112513 (PMC9697022; doi:10.3390/v14112513)
Supplement: Supplementary file 1 [file viruses-14-02513-s001.zip › viruses-2003296-supplementary.pdf]

Supplementary material

Title: Associations between NK Cells in Different Immune Organs and Cellular SIV DNA and RNA in Regional HLADR$^-$ CD4$^+$ T Cells in Chronically SIV$_{mac239}$-Infected, Treatment-Naïve Rhesus Macaques

Supplementary Figures and Tables

1 Supplementary Table

Table S1. The characteristics of SIV-infected Chinese rhesus macaques in this study.

Animal No.	Sex	Weight (kg)	Age (months)	Viral stock	Routine of inoculation	Viral quantification	Time of inoculation	Time of euthanasia
**G0101R**	F	4.15	49	$SIV_{mac}239$	Ir	100 TCID50	Jan 3, 2013	Jan 21, 2015
**G0102R**	F	4.25	51	$SIV_{mac}239$	Ir	100 TCID50	Jan 3, 2013	Jan 21, 2015
**G0104R**	F	4.55	54	$SIV_{mac}239$	Ir	100 TCID50	Jan 3, 2013	Jan 21, 2015
**G0105R**	F	4.8	47	$SIV_{mac}239$	Ir	100 TCID50	Jan 3, 2013	Jan 21, 2015
**G0106R**	F	3.9	55	$SIV_{mac}239$	Ir	100 TCID50	Jan 3, 2013	Jan 21, 2015

Ir, intrarectally; SIV, simian immunodeficiency virus; TCID, tissue culture infectious dose.

2 Supplementary Figures

Supplementary Figure S1

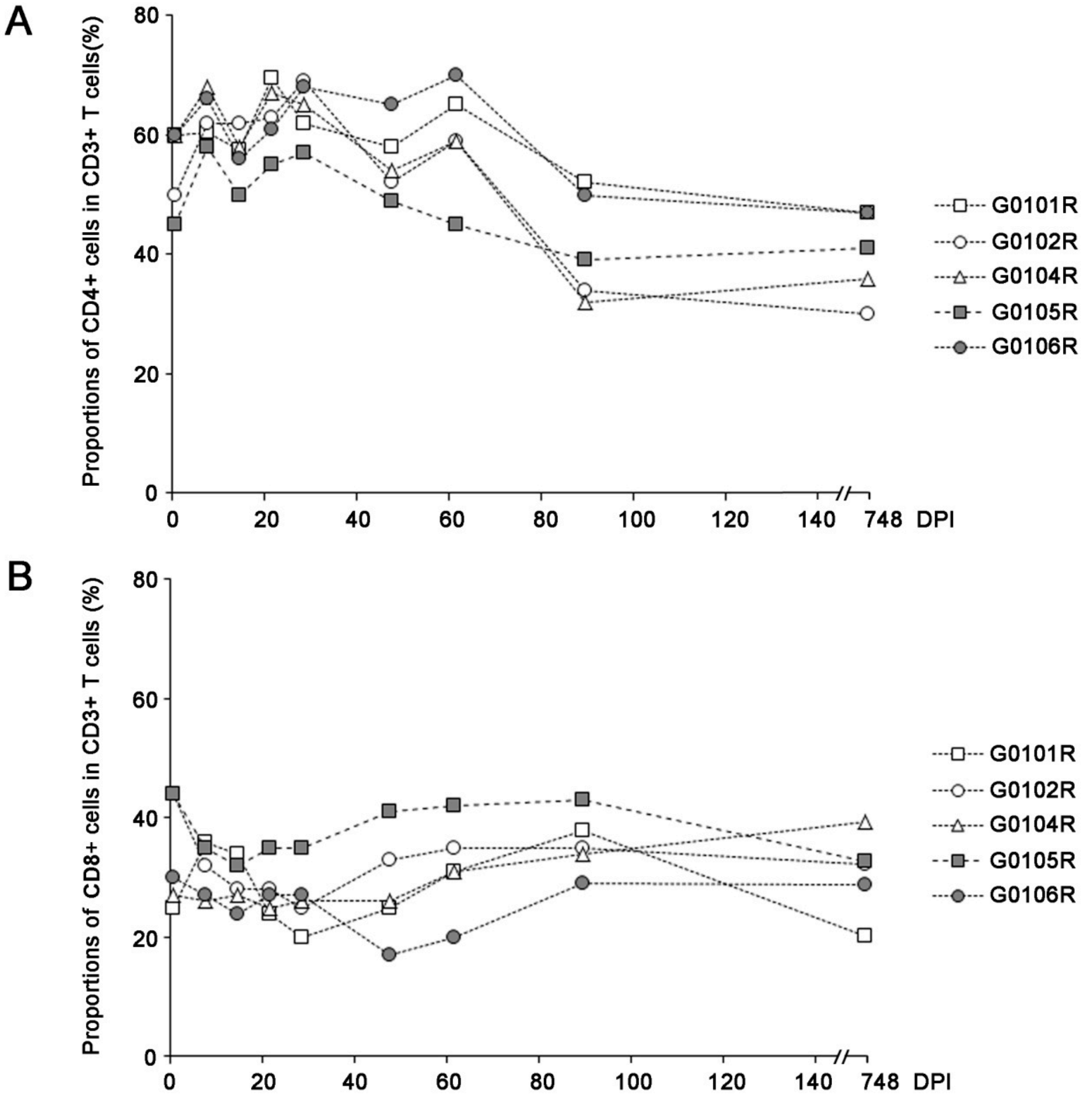

Figure S1. Dynamics of peripheral CD4⁺ T and CD8⁺ T cell counts in the bloodstream of 5 SIV-infected Chinese Rhesus macaques.

Supplementary Figure S2

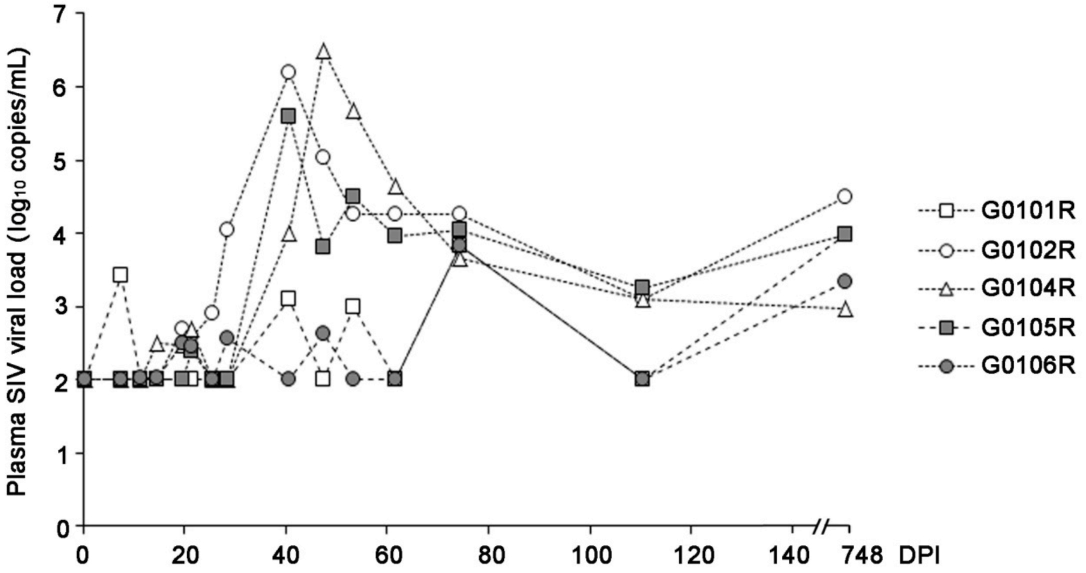

Figure S2. Dynamics of plasma SIV viral load in 5 SIV-infected Chinese Rhesus Macaques.
